# The Impact of Field Courses on Undergraduate Knowledge, Affect, Behavior, and Skills: A Scoping Review

**DOI:** 10.1093/biosci/biac070

**Published:** 2022-08-24

**Authors:** Xoco A Shinbrot, Kira Treibergs, Lina M Arcila Hernández, David Esparza, Kate Ghezzi-Kopel, Marc Goebel, Olivia J Graham, Ashley B Heim, Jansen A Smith, Michelle K Smith

**Affiliations:** Cornell University, Ithaca, New York, United States; Cornell University, Ithaca, New York, United States; Cornell University, Ithaca, New York, United States; Cornell University, Ithaca, New York, United States; Cornell University, Ithaca, New York, United States; Cornell University, Ithaca, New York, United States; Cornell University, Ithaca, New York, United States; Cornell University, Ithaca, New York, United States; Friedrich-Alexander-Universität, Erlangen, Germany; Cornell University, Ithaca, New York, United States

**Keywords:** field course, higher education, undergraduate, natural sciences, barriers

## Abstract

Field courses provide transformative learning experiences that support success and improve persistence for science, technology, engineering, and mathematics majors. But field courses have not increased proportionally with the number of students in the natural sciences. We conducted a scoping review to investigate the factors influencing undergraduate participation in and the outcomes from field courses in the United States. Our search yielded 61 articles, from which we classified the knowledge, affect, behavior, and skill-based outcomes resulting from field course participation. We found consistent reporting on course design but little reporting on demographics, which limits our understanding of who takes field courses. Cost was the most commonly reported barrier to student participation, and knowledge gains were the most commonly reported outcome. This scoping review underscores the need for more rigorous and evidence-based investigations of student outcomes in field courses. Understanding how field courses support or hinder student engagement is necessary to make them more accessible to all students.

Field courses in the natural sciences provide   immersive opportunities for students to leave the traditional classroom setting and experience natural phenomena outdoors. By engaging in experiential learning in the field, students gain valuable transferable skills in areas such as interpersonal communication, critical thinking, and the scientific process, along with gains for both conceptual knowledge and environmental literacy (Boyle et al. 2007, Fleischner et al. 2017, Scott et al. 2019, Race et al. 2021, Morales et al. 2022). Field courses also generate powerful affective outcomes that can support student success (Eiss and Harbeck 1969, Boyle et al. 2007). Previous research shows participation in field courses can encourage positive shifts in students’ science identity, sense of place, and self-efficacy (Chow and Healey 2008, Jolley et al. 2018). Likewise, field courses can inspire behavioral changes with long term implications, such as enrolling in science, technology, engineering, and math (STEM) courses, persistence in a STEM major, and pursuing STEM careers (Beltran et al. 2020, Halliwell et al. 2020).

Because of their great potential for increasing student success, field courses are taught in a variety of natural science disciplines, such as ecology and geosciences (Fleischner et al. 2017). Student participation in field courses is often listed as a key proficiency in natural science educational frameworks. For example, the Ecological Society of America's governing board elevated the importance of field-based natural history approaches to teaching ecology through the Four-Dimensional Ecological Education Curriculum Framework (Klemow et al. 2019), recognizing the general requirement for students to have field experience and be proficient with key field techniques.

Despite the increase in students taking courses in natural science disciplines, field course offerings have not increased proportionally to meet the demand (Smith 2004, Burke Da Silva 2014, Fleischner et al. 2017). The financial cost, rising university enrollments, and regulatory safety requirements make planning, organizing, and implementing field experiences logistically difficult (Burke Da Silva 2014). Narrative, descriptive literature reviews have linked field courses to positive cognitive, affective, and behavioral outcomes in the field of biology (e.g., Fleischner et al. 2017) and a systematic review of field course outcomes has been conducted in the geosciences (Donaldson et al. 2020). However, to our knowledge, no publications have systematically assessed how field courses affect undergraduate students across multiple courses, outcomes, and research studies in the natural sciences.

In the present article, we describe a scoping review that synthesizes the emerging body of evidence on participation in and outcomes for undergraduates in United States–based, natural science field courses. Scoping reviews are systematized approaches to synthesizing evidence that serve to determine the body of literature on a given topic and identify gaps in the literature and opportunities for growth. Specifically, we address three research questions: What internal and external factors are shown in the literature to affect participation in undergraduate field courses in the natural sciences? What knowledge, affective, behavioral, and skill-based outcomes result from participation in undergraduate field courses? And which study designs, methods, and measures have been used to study field courses? The resulting data can inform future research opportunities and improve institutional, departmental, and instructor-based practices for equitable field course design and evaluation.

## Study approach

**Figure 1.**
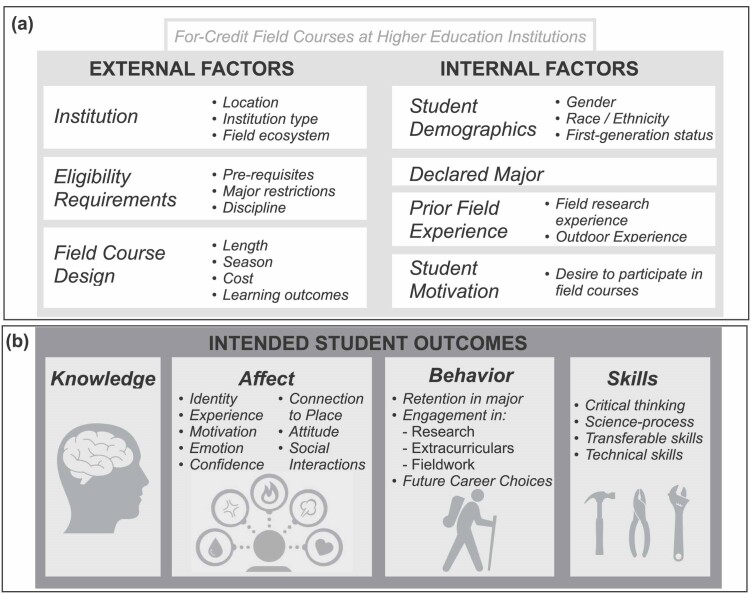
The conceptual framework for this study situates student outcomes—specifically, knowledge, affect, behavior, and skill-based outcomes—as a result of participation in field experiences as dependent on external and internal factors.

To address our three research questions, we created a conceptual framework a priori for our scoping review, which guided our literature search and data extraction and provided a scaffold outlining the external and internal factors that might influence student outcomes in field courses (figure 1).

The external factors encompassed field course institution characteristics including field locations and course design factors such as the number of days in the field and the season. We also documented any explicit or implicit barriers to participation, including financial costs (i.e., course fees) and course length requirements, which both affect student participation, as well as student outcomes (Morales et al. 2022)

Our conceptual framework also recognizes the importance of internally student-held factors such as demographics (i.e., gender, race or ethnicity, first-­generation status) and prior field experience. Such factors have been shown to influence student affective domains, including their attitudes toward field work (Boyle et al. 2007) and motivation, which has been shown to affect student participation in field courses (Scott et al. 2019).

### Scoping review methodology and protocol preregistration

We followed the Preferred Reporting Items for Systematic Reviews and Meta-Analyses (PRISMA) Extension for Scoping Reviews (Tricco et al. 2018) checklist and guidelines to ensure a robust and replicable scoping review process (Levac et al. 2010, Tricco et al. 2018). The protocol for this scoping review was preregistered on the Open Science Framework and is available at the following address: https://osf.io/qxkhm. The scoping review included seven phases: designing a literature search protocol, running a search of databases according to that protocol, identifying citations of interest, screening manuscript titles and abstracts, screening the full text of manuscripts, extracting the data from the remaining manuscripts, and analyzing of extracted data. The PRISMA flow diagram (supplemental figure S1) summarizes the process.

### Databases, search methods, and citation management

**We developed and** tested a comprehensive search strategy to identify all available research pertaining to the three primary research questions. The search items included variations on the terms *field course*, *undergraduate*, and *natural science* (supplemental table S1). The searches were performed on 8 April 2020 in the following electronic databases: the Web of Science Core Collection (accessed via Web of Science), CAB Abstracts (accessed via Web of Science), and the Education Resources Information Center (accessed via EBSCOhost). We searched all relevant gray literature sources for documents published outside of peer-reviewed journals: ProQuest Dissertation and Theses (ProQuest), the Science Education Research Center, *CourseSource*, and the QUBES (Quantitative Undergraduate Biology Education and Synthesis) Hub. We also performed a hand search of the *American Biology Teacher*, screening the table of contents from volume 81, issue 3, to volume 82, issue 3, against the predetermined criteria for inclusion. The search strategies are accessible at the following address: https://osf.io/hf9vx.

We defined the key terms to inform the search strategy, selection criteria, and data extraction, which were integral to bounding and answering our three research questions (supplemental table S2). Particularly, field courses were considered credit-based full-term courses where undergraduate students leave the classroom and interact with the outdoor environment at least once. These classes occurred in the natural sciences, which we defined as disciplines that study natural events using scientific methods (Ledoux 2002).

### Eligibility criteria and study selection

The PRISMA flow diagram (figure S1) shows the study selection process and indicates the number of articles excluded at each phase of screening. We included original peer-reviewed published articles if they met a predefined set of nine eligibility criteria (box 1). The eligibility criteria were created on the basis of the characteristics of our research questions. Specifically, we focused on United States–based institutions so that we could provide action-oriented research for US institutions. We also restricted our search to studies published in the 2000s, to focus on practices for improving enrollment and participation in today's environment. We excluded any articles that did not meet all of our criteria.

**Table 1.** Description of study designs used in scoping review articles and rankings of overall study rigor.

	Overall		Pre- and posttreatment assessment		Control or comparison group			Within assessment type	
Assessment type	Number	Percentage	Number	Percentage	Number	Percentage	Overall study rigor	Number	Percentage
Mixed	33	54	18	30	6	10	More rigorous	9	27
							Moderately rigorous	13	39
							Less rigorous	11	13
Quantitative	9	15	5	8	3	5	More rigorous	3	33
							Moderately rigorous	2	22
							Less rigorous	4	44
Qualitative	19	28	1	2	0	0	More rigorous	1	5
							Moderately rigorous	4	21
							Less rigorous	14	74
*n*	61		24		9		–

**Box 1. Eligibility criteria for article inclusion in the scoping review.**

An article should be focused on higher education institutions. It should be focused on universities based within the United States but including study abroad programs. It should be focused on undergraduate students taking a for-credit field course. It should be focused on classes that occur outside the classroom in the field. It should be focused on classes in the natural sciences. It should contain an evaluation of factors influencing undergraduate participation or persistence, or student outcomes (e.g., knowledge, affective, or behavioral outcomes). It should have been published between January 2000 and April 2020. It should describe original research that employed qualitative or quantitative research methods. Finally, it should be written in English.

All of the present coauthors participated in the article screening stage. Prior to study selection, a screening protocol was piloted on five articles to ensure consistency among raters. Titles and abstracts were screened independently by pairs of coauthors, using Covidence systematic review software (Veritas Health Innovation, Melbourne, Australia; available at www.covidence.org), according to the eligibility criteria. Then, full-text screening was performed independently by pairs of coauthors to exclude articles that did not meet all inclusion criteria. Any disagreements were resolved by an independent third coauthor.

### Data extraction and analysis

Data extraction was independently completed by pairs of coauthors with a focus on five main categories: study design (supplemental table S3), external and internal factors affecting participation (supplemental tables S4 and S5), field course design (supplemental table S6), and student outcomes (supplemental table S7). Any discrepancies between pairs of coauthors were resolved through discussion to consensus.

As part of extracting data about study design, all of the coauthors individually scored articles for their methodological and analytical rigor on a scale of *more rigorous*, *moderately rigorous*, and *less rigorous* (table S3). These rankings were then discussed within the pairs of coauthors. For methodological rigor, the criteria included ranking the clarity of the sampling methods and whether the sampling strategy was suitable for the study design. For analytical rigor, the criteria included whether the analysis was clearly described, there was an appropriate level of detail to interpret study results and repeat the study, and the analysis maximized the chance of producing data with discernible patterns. The authors included comments justifying their decisions about each study's methodological and analytical rigor (e.g., the study did not describe the process of selecting illustrative quotes from the student surveys).

The final study rigor score was a combination of scores for both methodology and analysis. If the methodology and the analysis were both ranked as more rigorous, then the study received a more rigorous score overall. If the study's methodology and analysis were both ranked as less rigorous, then it was ranked less rigorous overall. If the study's methodology and analysis were mixed between two different levels of rigor (e.g., a more rigorous methodology and a less rigorous analysis or a less rigorous methodology and a moderately rigorous analysis), then it ranked as moderately rigorous. After the ranking was complete, we also categorized the type of student outcome data described in each study (e.g., surveys), whether or not research questions or a hypothesis was included in the manuscript, the total types of data cited as evidence of student outcomes, and whether the article included a discussion of study limitations. These measures allowed us to gain a detailed understanding of current methods and associated rigor of research on undergraduate field courses.

## Findings

In total, 61 articles were included in the systematic scoping review (supplemental table S8). The number of publications increased over time, ranging anywhere from three to eight articles each year between 2010 to 2019 (supplemental figure S2). The majority of articles were peer reviewed (*n* = 46). In addition, there were 10 theses or dissertations, three reports, and two book chapters.

### What internal and external factors affect ability and motivation to participate in undergraduate field courses in the natural sciences?

Using the conceptual framework described in figure 1, we investigated the external and internal factors that influence student outcomes in field courses. Following our literature search, we extracted data about these factors, along with any reported data about student outcomes of field courses.

**Figure 2.**
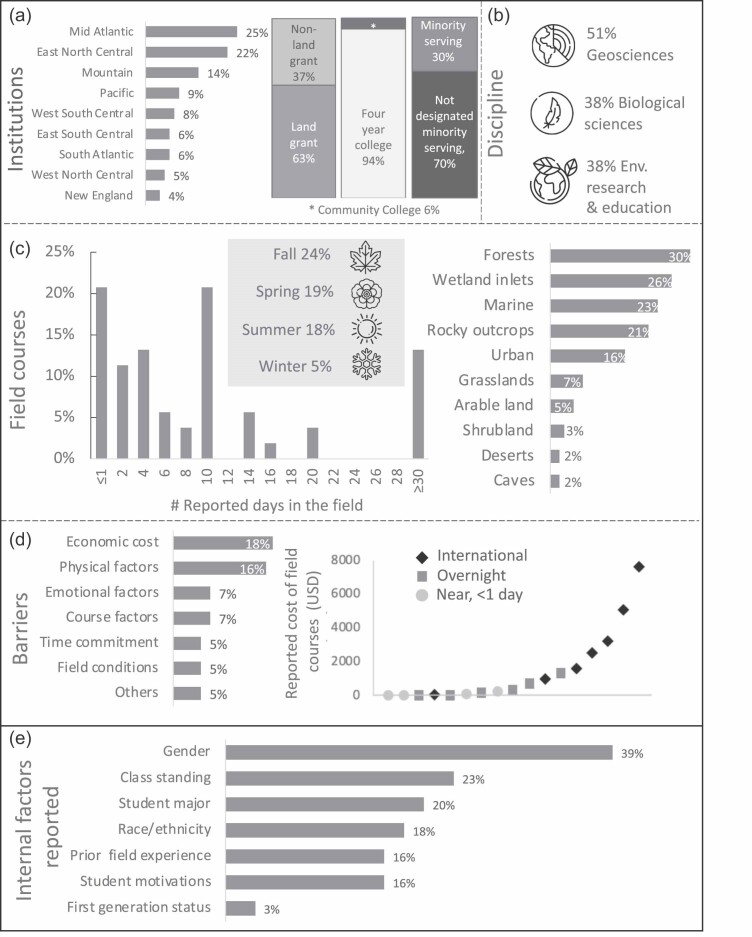
Field courses characterized by the reported (a) institutional location; (b) natural science discipline; (c) days spent in the field, seasons offered, and study areas; (d) student barriers; and (e) internal student factors reported.

The articles depicted 79 different field courses representing 61 different US institutions (figure 2a and supplemental figure S3). Ten articles investigated field courses offered by United States–based institutions that took place abroad (*n* = 6, Costa Rica; *n* = 3, Ecuador; *n* = 1, Honduras). Of the 79 field courses referenced within our data set, most were based at public (*n* = 57), land-grant universities (*n* = 50). Only four of the referenced institutions were community colleges (figure 2a). Twenty-four of the institutions were considered to be minority serving, including emerging minority-serving, Hispanic-serving, and tribal colleges or universities.

Geosciences was the most frequently represented discipline in our data set (*n* = 31), with fewer articles about field courses in biological sciences (*n* = 23) and environmental research and education (*n* = 7; figure 2b). Using National Science Foundation (NSF) discipline division designations, 21 articles were in the Earth sciences, 15 in environmental biology, 8 in the dynamics of socioenvironmental systems, 8 in integrative organismal systems, 1 in the ocean sciences, and 1 in atmospheric and geospace science education. Seven additional articles were in disciplines that did not match an NSF division (e.g., environmental humanities).

Out of the 61 articles, 54 documented the time students spent in the field per semester. The majority of field courses (*n* = 29 articles) spent 10 days or less in the field (figure 2c). Only a few courses (*n* = 7) spent more than 30 days in the field as part of a semester-long course.

The season in which field courses occurred was well documented among the articles in our data set, with 56 recording this information. Of the articles that reported the season, 23 described spring courses (March to May), 19 fall courses (September to November), 16 summer courses (June to August), and two winter courses (December to February; figure 2c). The remaining papers described courses that were offered during multiple overlapping seasons or terms.

Almost every article (*n* = 58) reported information about the type of field ecosystem that the course focused on. Most of the reported field experiences occurred in natural areas (*n* = 56 articles). The other areas that were mentioned included informal educational settings (e.g., zoos, museums), Native American reservations, research and breeding facilities, and farms. The articles that reported field ecosystem type were focused on forests (*n* = 17) and wetland inlets, including rivers (*n* = 15), marine (*n* = 13), and rocky geologic outcrops (*n* = 12; figure 2c).

Less than half (*n* = 24) of the articles in our data set reported barriers to participation in field courses. Of all the articles in our data set, economic cost was most frequently reported, described in 11 articles (figure 2d), followed by physical factors (*n* = 10), such as exhaustion (e.g., atypical sleeping conditions) or preexisting disabilities. Other barriers were reported in less than 10% of the articles, including emotional factors, course factors, time commitments, and field conditions. Where they were reported, the course fees for the students ranged from free to $7531 per student. The costs were greater for overnight and international experiences than for nearby field experiences.

The internal student factors (figure 1) were infrequently reported among the articles in our data set (*n* = 24). Gender was the most commonly reported student factor in the field courses (figure 2e). Across the 1616 total students with reported gender, 55% of those students were female, and 45% were male. Only 11 articles reported race or ethnicity data on the basis of a total of 948 students enrolled in field courses. Within the articles that reported these data, 81% of the students identified as White, and 19% identified as a race or ethnic group other than White. Only two articles reported data on first-generation status of students in field courses, and one article included specific counts of students who identified as first generation or low income (35%, *n* = 13 out of 37 students).

Similarly, few articles (*n* = 17) reported student class standing. Of the 445 students represented in those articles, 37% were first-years and 27% were seniors. Several articles reported class standing data in aggregate (e.g., combining groups); therefore, the remaining students were either sophomores, juniors, graduate students, or nontraditional students. Student majors were reported in 12 articles of our data set. Because the names of majors are inconsistent across institutions, it was difficult to accurately document how many students were enrolled in distinct majors; however, the majority of the students majored in geology, biology, or related fields. A list of all of the majors is in the supplemental material (supplemental table S9).

Whether or not students had previous field experience was infrequently reported (*n* = 10), although these data varied widely across articles; most were focused on previous field experience that related to specific outdoor skills students had prior to entering the field course (e.g., swimming, farming) rather than prior experience with field research.

Of the 11 articles that assessed student motivations to participate in field courses, five were focused on assessing learning motivations, four on assessing programmatic requirements, four on assessing career skills, three on assessing research skills, three on enjoyment or wellness, two on being outdoors, and one on social motivations. In only one article was the association between student internal motivations and higher field course grades evaluated (Dykas and Valentino 2016).

### What knowledge, affective, behavioral, and skill-based outcomes result from participation in undergraduate field courses?

**Figure 3.**
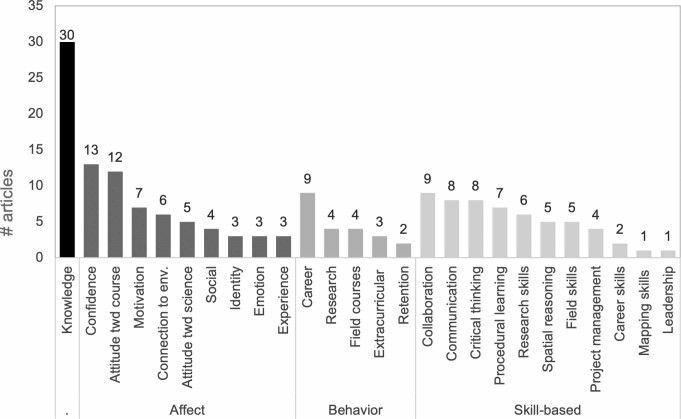
Reported student outcomes as a result of participation in field courses, grouped by knowledge, affect, behavior, and skill-based outcomes.

Following our literature search and data extraction, we classified the intended student outcomes into four categories, including knowledge, affect, behavior and skill-based outcomes (figure 3).

We found 30 articles assessing knowledge outcomes including factual and conceptual understanding, metacognition, and concept retention (figure 3). The majority (*n* = 14) collected knowledge data through instructor-designed assessments. Additional articles used student self-assessments of knowledge gains (*n* = 11) or both instructor-designed assessments and student self-assessments (*n* = 5). Most of the articles (*n* = 36) with empirical evidence identified at least one positive and statistically significant association between field course participation and knowledge outcomes. Only two articles reported neutral knowledge outcomes—that is, knowledge neither increased nor decreased. One article reported both positive and negative knowledge outcomes depending on the parameter.

We found 29 articles focused on affective outcomes, with as many as five affective outcomes reported within a single article. Articles reporting on student affect most frequently focused on confidence and attitudes toward the course. Twenty-eight articles empirically identified at least one positive and statistically significant association between field courses and affective outcomes. Three articles reported neutral outcomes on affect, three reported both positive and negative outcomes, and one article reported negative affective outcomes in attitudes toward science.

Only 18 articles focused on behavioral outcomes, with as many as three behavioral outcomes reported in a single article. The articles reporting on behavior were most frequently focused on current or future careers as a result of field course participation (*n* = 9). Seventeen articles empirically identified at least one positive and statistically significant association between field courses and behavioral outcomes. One article reported both positive and negative behavioral outcomes.

Skill-based outcomes were reported in 28 articles, with as many as five outcomes reported in a single article. We found that transferable skills such as collaboration, communication, and critical thinking were more commonly assessed. All 28 articles empirically identified at least one positive and statistically significant association between field courses and skill-based outcomes.

### Which study designs, methods, and measures have been used to study undergraduate field courses?

The majority of the articles in our data set used a mixed methods approach (*n* = 33; table 1). More than half of those used pre- and posttreatment assessments, and few (*n* = 6) provided a control or comparison group. Similarly, few (*n* = 9) articles used assessments with validity evidence designed to measure knowledge, affect, or skill-based outcomes for undergraduate students (supplemental table S10). Finally, some articles within our data set used purely qualitative methods (*n* = 19; e.g., interviews, written reflections, focus groups).

Overall, we classified most studies as either less rigorous (*n* = 29) or moderately rigorous (*n* = 19; table 1). For example, 20 of 61 articles included only anecdotal data (e.g., quotes selected without systematic analysis) as evidence of learning outcomes, rather than including empirical data obtained through observation, documentation, and student assessment or using rigorous qualitative methods (e.g., Saldaña 2015). Only 13 of the reviewed articles were considered more rigorous (i.e., clearly described and with a scrupulously and meticulously carried out study design and analysis; Allen 2017). The most commonly used data type for assessing student outcomes is surveys, followed by course assessments, student field notes or reflections, and direct observation by researchers (supplemental figure S4a). We found that more than half of the articles (*n* = 35) included a clearly stated research question or hypothesis, whereas others (*n* = 26) lacked a research question or hypothesis (figure S4b). In terms of citing evidence for student outcomes, just over half of the articles (*n* = 34) relied on only one or two types of data. Finally, over half of the articles (*n* = 36) reflected on their study's limitations.

## Interpreting our scoping review findings

The primary goal of this scoping review was to identify how internal and external factors contributed to student learning outcomes in natural science field courses in United States–based institutions, on the basis of available evidence (figure 1). This work compliments a recent study that used inputs from experts in undergraduate field education and STEM learning and a basic literature review to present a model that characterizes student outcomes in field courses and field-based research experiences (O'Connell et al. 2022). The student outcomes are a function of student context factors (e.g., identity, prior knowledge, and skills) and field course design (e.g., setting, social interactions). Related work has identified field course design features, instructional strategies, and desired student outcomes by surveying field experience program directors, instructors, and coordinators (O'Connell et al. 2021).

Unlike these existing studies, our study independently converges on themes that emerged from a systematic synthesis of published research on the deeper focus of field courses with a review of the methodological and analytical rigor of each article. For example, as other models have, we focus on identifying what external factors (e.g., field course design factors) and internal student-held factors (e.g., prior field course experience) influence student outcomes (figure 1). However, by surveying existing literature on field courses, our research has uncovered a range of immutable factors that our framework recognizes such as student demographics or slow-to-change factors such as institutional minority serving status, which collectively affect student participation in field courses. In light of these findings, future studies should empirically test the role of specific factors such as these within the context of existing models for the outcomes of field experiences (O'Connell et al. 2022).

These future studies are important because field courses have the potential to provide equitable opportunities for underrepresented and minoritized (URM) students, by improving retention in the major, increasing GPAs at graduation, and improving college graduation rates (Beltran et al. 2020). These improvements are notable because racial, ethnic, and economic disparities hamper STEM representation of undergraduate URM students in the United States (NASEM 2011, Holman et al. 2018). National data show that the disparities for URM students in comparison to White and Asian students increase at each STEM degree level—for example, from bachelor’s to master’s degrees (Estrada et al. 2016). Some disciplines, such as geosciences, have seen little to no improvement in ethnic and racial diversity at multiple degree levels over the past 40 years (Bernard and Cooperdock 2018). Studying the ways field courses can be used to support students from many backgrounds has the potential to have a profound impact on the natural science field.

### Knowledgefocus overshadows affective, behavioral, and skill-based outcomes

We found that research on undergraduate learning outcomes from field experiences has been overwhelmingly focused on knowledge gains, with less attention paid to student affect, behavior and skill-based outcomes ­(figure 3). Although institutional factors, field course design, and student factors have implications for student outcomes ­(figure 1), more research is needed on the causal mechanisms behind them. In the present article, we highlight a need for extending assessment of field courses to include behavioral and affective outcomes, make evidence-based recommendations for rigorous future research, discuss important barriers for student participation, and propose future steps to address reported barriers to participation in field courses.

Our research reflects recent findings that field course instructors have predominantly been focused on assessing knowledge based outcomes (O'Connell et al. 2018). The results may be because assessing knowledge is relatively familiar and easy to collect; knowledge-based outcomes are traditionally valued more than affective, behavioral, or skill-based outcomes in higher education; and field experiences are increasingly recognized for providing unique knowledge gains.

Field courses provide a wide spectrum of key proficiencies outside of knowledge including career choices, leadership skills, and critical thinking (e.g., Peacock et al. 2018, Peasland et al. 2019, Scott et al. 2019). Field courses can also provide important affective outcomes such as fostering connections with the environment and a sense of place, both of which can strengthen students’ intrinsic motivation to learn (Jolley et al. 2018). Therefore, to address this scarcity of attention to noncognitive outcomes of field courses in the literature, we call for increased collaboration between instructors and education researchers to track how participation in field courses affects student outcomes in the affective, behavioral, and skill-based learning domains. For example, networks such as the Undergraduate Field Experiences Research Network (www.ufern.net) connect field practitioners and education researchers through collaborative projects that are supported by network meetings, working groups, and workshops (O'Connell et al. 2018). A recent workshop focused specifically on affective outcomes (Ward et al. 2021). Such networks are centers for research excellence, idea generation, and academic support for those involved in field experiences.

### The lack of standardization and analytical rigor is an opportunity for future research

In addition to the relative scarcity of data about noncognitive outcomes of field courses, there was limited evidence in this review of hypothesis-driven analytical articles with methodological and analytical rigor that tested for associations between field course participation and student outcomes (table 1, figure S4). These results pose a constraint to generalizing about the mechanisms connecting course activities and outcomes, results that are consistent with previous research that investigated geoscience field courses (Mogk and Goodwin 2012).

We recognize that many studies in our data set can be classified as hard-earned practitioners’ wisdom. Indeed, the limited evidence in these studies does not diminish the obvious importance of field courses for student outcomes but, instead, emphasizes the need to improve and expand on established research using rigorous methods to demonstrate the complex mechanisms leading to student outcomes. By adopting more rigorous student assessment practices (e.g., using a codebook and multiple raters to analyze qualitative data) that span multiple domains of learning, instructors will more clearly demonstrate the unique contributions that field courses have within the curriculum. Professional expertise from university teaching and learning centers may be essential to supporting field course instructors who wish to conduct rigorous education research. Ultimately, the onus of responsibility should lie with institutions to develop, fund, and recruit field course instructors to participate in professional development opportunities led by education ­researchers (Diaz-Martinez et al. 2019).

Given the lack of standardized data collection and analytical methods within the articles included in this scoping review, we envision an opportunity for the field research education community to bring increased rigor and broader assessment goals to research on field courses. For example, rigorous longitudinal and experimental designs can be implemented using validated assessments (e.g., the pre- and posttreatment attitudinal surveys described in Simmons et al. 2008) to understand external and internal student-held factors that affect field course participation and student outcomes. Alongside experimental designs, we encourage the use of thick descriptions of field course design elements, intended learning outcomes, assessment practices, and student demographics (e.g., Scott et al. 2019) as a means of establishing external validity in a study that includes qualitative data (Geertz 1973). These descriptions provide context for researchers and instructors to make connections between published data for their own courses, as well as to provide raw data for future reviews.

### Field courses have unique barriers constraining participation

The results from this review underscore the unique challenges that undergraduates may face when participating in field courses, including economic cost, the time required off campus, and physical burdens (figure 2d). Our results are consistent with recent research from a nationwide undergraduate student survey, which showed that income was proportionally the most frequent barrier to student participation in field experiences (Jensen et al. 2021). Eighty percent of the undergraduates at US postsecondary institutions work while they are enrolled full time in classes, so the financial burden of taking a field course can extend beyond field site travel costs to include wage losses for employed students (NCES 2002). Such financial costs have cumulative and compounding impacts that limit access to field course opportunities. One possible solution is for institutions to offer funding for scholarships to offset costs for students. Curricular budgets in the natural sciences could explicitly allocate funding to the field experiences required within educational frameworks.

To address the challenges students face beyond course costs, we recommend that future studies collect more data on the barriers to participation in field courses and examine how early interventions could address the external and internal factors that promote and inhibit participation in field courses. For example, future studies could explore the effectiveness of interventions that set early expectations on how to adequately prepare for field conditions with appropriate gear (University of California 2019) and address uncertainty about restroom access. These challenges may be especially significant barriers to students without prior outdoor experience and could be contributors to exclusion and subsequent attrition of URM students from field courses and STEM fields more broadly. In order to reduce exclusion and attrition, we also encourage the development, implementation, and assessment of interventions geared toward promoting inclusive field practices and practicing cultural sensitivity. Although field courses can be critically important for building cultural sensitivity and inclusive student experiences (Nieto 2006), such topics were not formally assessed as outcomes within our data set.

Barriers may be experienced differently depending on student-held characteristics such as demographics and prior experience, but they were infrequently reported within our data set (figure 2e). For studies that reported internal factors, gender was most commonly reported, but race or ethnicity, first-generation status, low-income status, and prior field experience were rarely reported. Reporting internal data can help identify areas where field course improvements are needed, in particular to better support students historically excluded in STEM (Giles et al. 2020). Moving forward, research should consider a broad range of internal factors, not just demographics but including worldview, interests, identity, personal needs, and prior experience (O'Connell et al. 2022). Understanding factors such as students’ prior experience can be important for instructors to gauge the students’ level of familiarity and comfort learning within a natural environment and for researchers to more accurately assess the outcomes of field courses (Mogk and Goodwin 2012).

One promising discovery of our review was that minority serving institutions (MSI) and emerging MSIs, which represent only 14% of degree-granting, Title IV–eligible higher-education institutions in the United States (NASEM 2011), represented 30% of the courses reported on in our data set (figure 2a). MSIs are essential for providing historically excluded students with field courses that are thoughtfully designed to be safe spaces to learn about the natural sciences in the field. More rigorous and systematic reporting of internal student held factors is still needed to understand how to improve participation in field courses.

Compared with underreported student factors, details about field course context and design were provided by most articles. For example, the majority of classes described in our data set took students off campus for a day or more (figure 2b). Despite the clear benefits of multiday or -week immersive experiences, this schedule poses a participation barrier to some students with limited time to devote to a single course. One possible solution that reduces this barrier is for the instructors to offer and evaluate the efficacy of shorter field experiences that occur on or near campus within class time. Effective learning experiences can occur in urban or suburban areas (e.g., Kirkby 2014) at on-campus or near-campus locations (e.g., parks, natural areas), which are more easily accessible for students and address a growing student interest in studying ecosystems with significant human impacts (Fleischner et al. 2017). Field course designs with reduced time commitments outside of class time may increase accessibility for students and reduce the overall effort, time, and financial costs to students, instructors, and the institution. More research is needed, however, to differentiate the impact of short-term experiences with long-term immersion experiences to identify shared and unique benefits or costs to students, instructors, and institutions.

## Future directions

This systematic scoping review reveals the emerging body of field course research in the natural sciences, emphasizing the importance of understanding how both external and internal factors affect student outcomes. The articles included in our review were predominantly focused on field course design, largely omitting internal student factors. Access to and inclusion within field courses continue to be major concerns that instructors and administrators must address, particularly for students that are historically excluded because of race or ethnicity, disability, or first-generation or socioeconomic status. Moving forward, field course research must prioritize accessibility and inclusion, and that includes reporting data on student factors such as demographics and prior experience and assessing student affective and behavioral outcomes, which were underreported. Researchers can support instructors by addressing their urgent need for rigorous hypothesis-driven analytical studies that identify and assess factors that enable or constrain student success in field courses. Improving the rigor and broadening the scope of field course research will be critical to the design of feasible, appropriate, and effective interventions to improve undergraduate student outcomes in the natural sciences.

## Supplementary Material

biac070_Supplemental_File

## Author Biographical

Xoco A. Shinbrot is a conservation social scientist, Kira Treibergs is a postdoctoral scholar, Kate Ghezzi-Kopel is a health sciences and evidence synthesis librarian, and Marc Goebel is a senior lecturer and research associate in the Department of Natural Resources and the Environment at Cornell University, in Ithaca, New York, in the United States. Lina Arcila Hernández is a postdoctoral scholar, David Esparza is a PhD candidate, Olivia J. Graham is a PhD graduate, and Ashley B. Heim is a postdoctoral scholar in the Department of Ecology and Evolutionary Biology at Cornell University, in Ithaca, New York, in the United States. Jansen A. Smith is a postdoctoral researcher in the Department of Geography and Geosciences at Friedrich-Alexander-Universität in Erlangen, Germany. Michelle K. Smith is a professor of biology in the Department of Ecology and Evolutionary Biology and a senior associate dean for undergraduate education in the College of Arts and Sciences at Cornell University, in Ithaca, New York, in the United States. All of the authors were affiliated with Cornell University at the time of research.
