# Supplementary material for: The Impact of Field Courses on Undergraduate Knowledge, Affect, Behavior, and Skills: A Scoping Review
Source: Bioscience. 2022 Aug 24;72(10):1007–17. doi: 10.1093/biosci/biac070 (PMC9525126; doi:10.1093/biosci/biac070)
Supplement: biac070_Supplemental_File [file biac070_supplemental_file.docx]

# FIGURES

#### **Figure S1.** PRISMA Flow Diagram

*
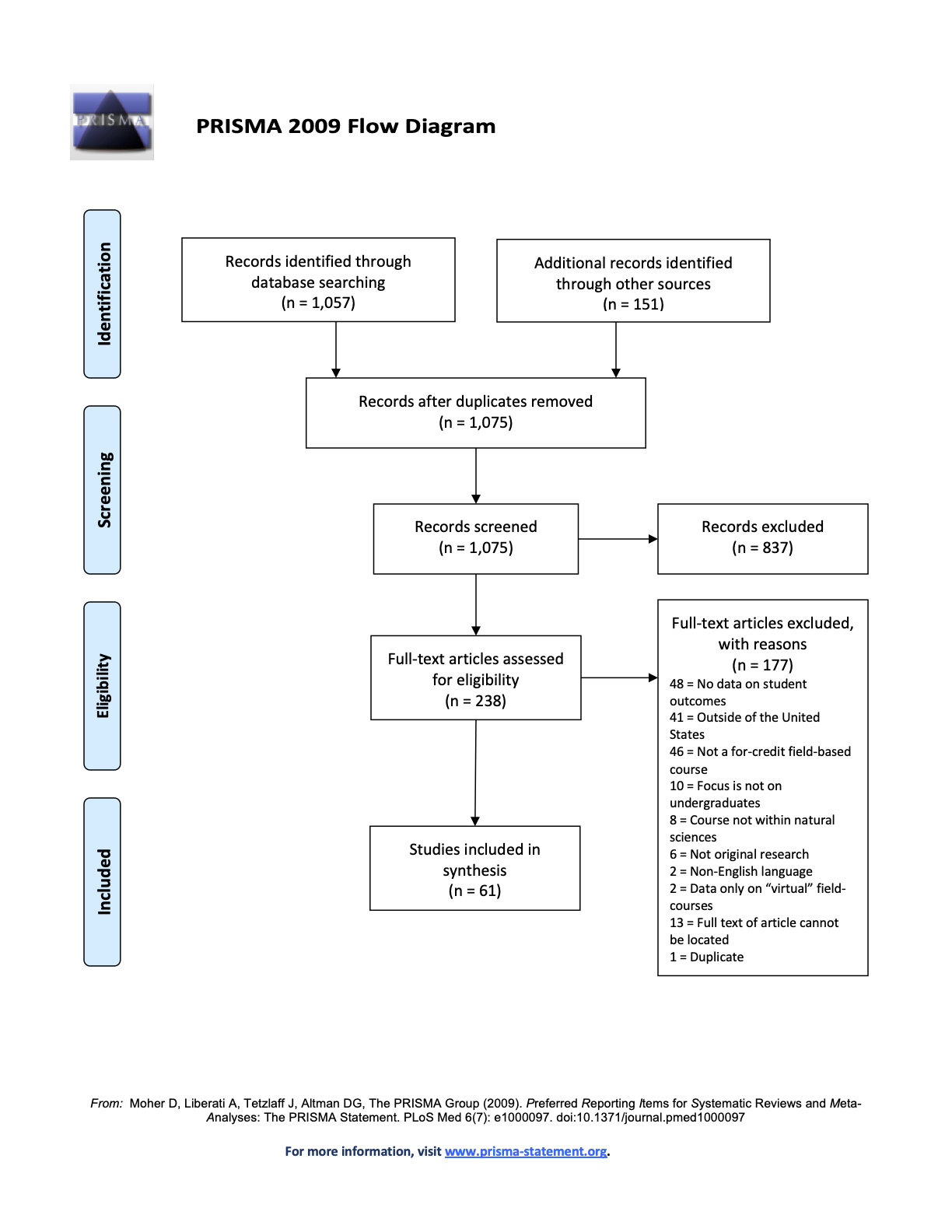
*

#### **Figure S2.** Number of articles published per year on US-based field courses in the natural sciences.


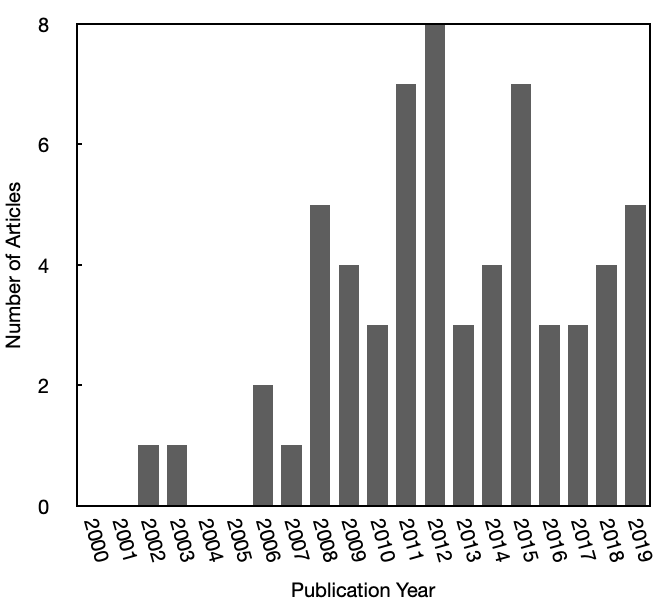


#### **Figure S3.** The geographic location of institutions with published field courses and associated field sites (ArcGIS Pro 2.6.1.). If the institution has no connecting line to a field site or the institution has no “Field Sites Near Institution” label, information about a field site was not provided or the field course stayed on campus.


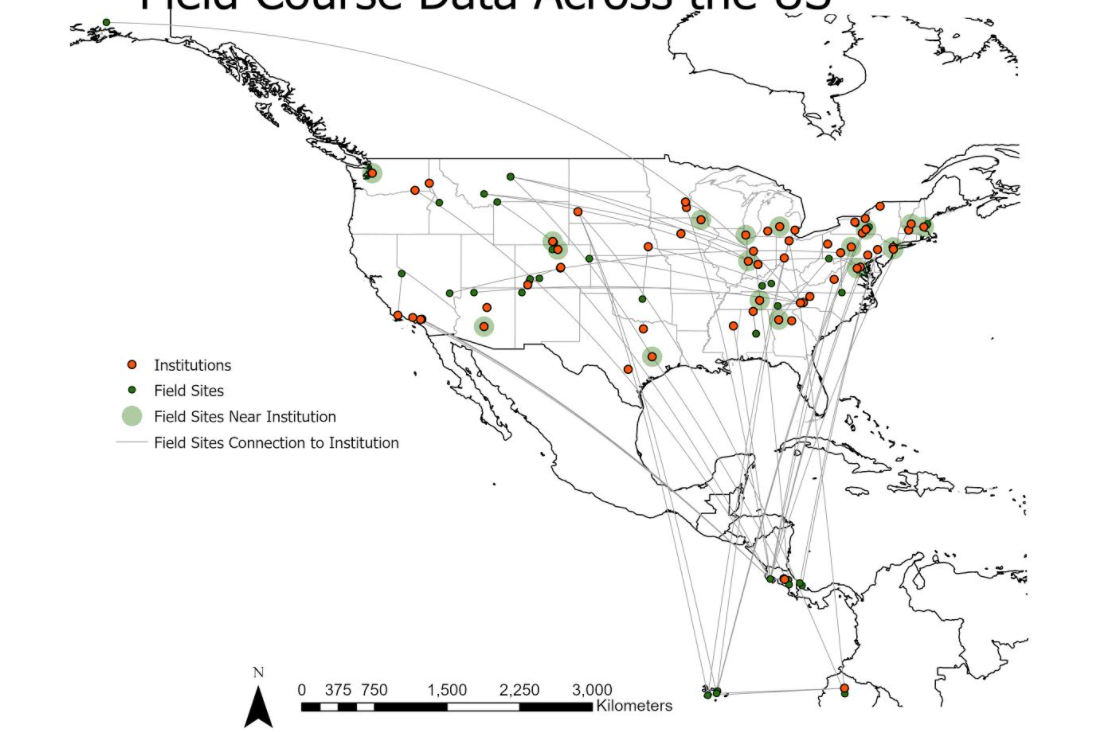


#### **Figure S4.** **Study Rigor.** a) The articles in our dataset assessed student outcomes of field courses, while citing a variety of evidence types (note that categories are not mutually exclusive, as single studies can contain multiple data types). b) Based on coder consensus (as described in section *3.4 Data extraction and analysis*), articles were classified as being ‘more rigorous,’ ‘moderately rigorous,’ and ‘less rigorous’. Included in this assessment was whether the article included clearly defined research questions or a hypothesis, the number of types of student outcome data, and whether the article included a discussion of the limitations of the study. Values superimposed on bars indicate the number of articles in each category.


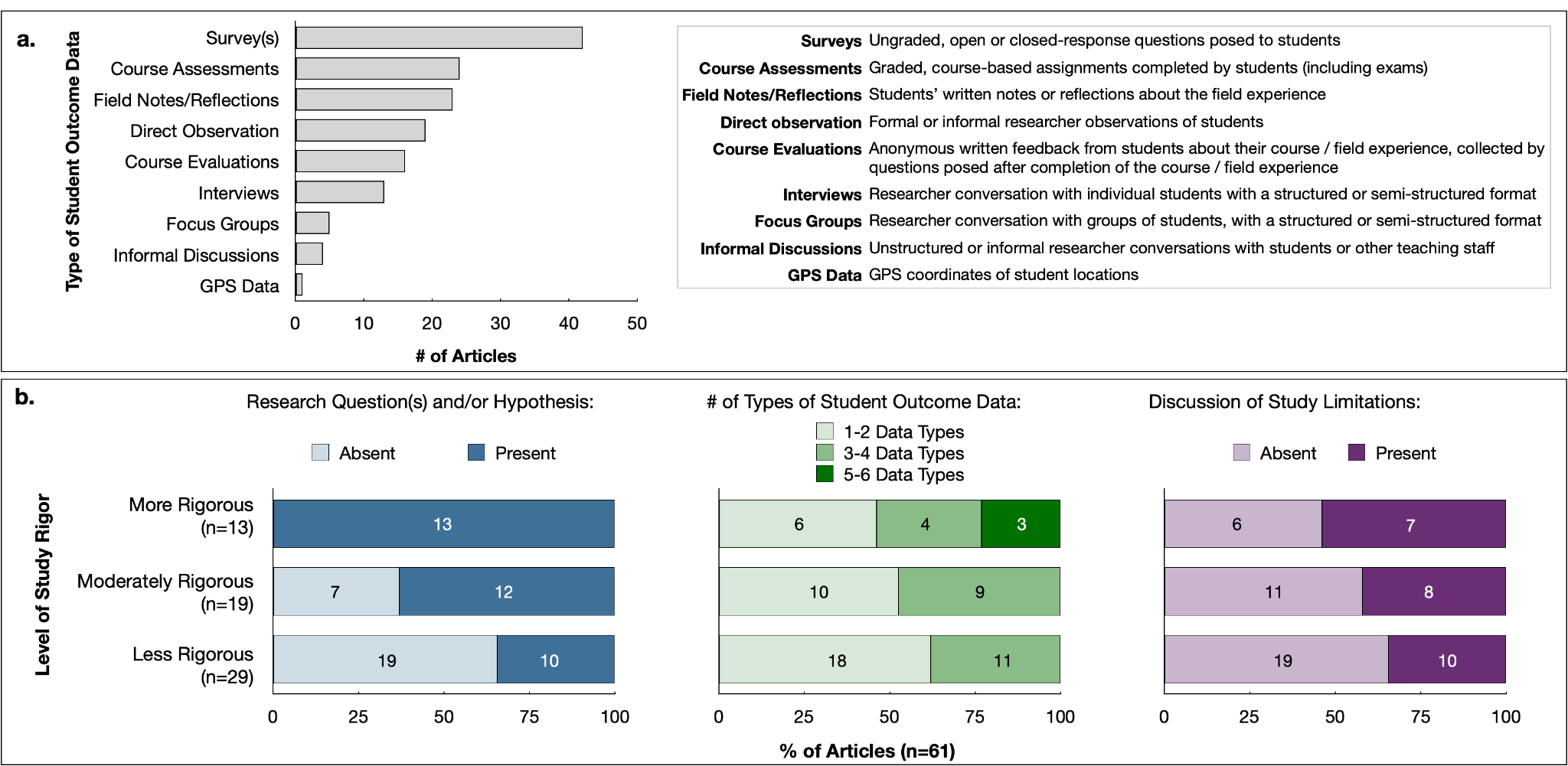


# TABLES

#### **Table S1.** Search strategy for Web of Science Core Collection (via Web of Science platform)

| 1. TS=("field course*" OR "field stud*" OR "field research" OR "field immersion" OR "field camp*" OR "field trip*" OR "field-trip*" OR "fieldtrip*" OR "field school" OR "fieldschool" OR "field work" OR "fieldwork" OR "field experience*" OR "field practice" OR "field-based" OR "field based" OR "place-based*" OR "place based*" OR "out-of-school learning" OR "outdoor experien*" OR "outdoor education*" OR "learn outdoors" OR "outdoor learning" OR "outdoor class*" OR "outdoor course*" OR "outdoor school*") |
| --- |
| 2. TS=(undergrad* OR "college student*" OR "college class*" OR "college course*" OR "college education" OR "university student*" OR "university class*" OR "university course*" OR "university education" OR "higher education" OR bachelor* OR "post-secondary" OR "four year institution" OR "four year collegiate institution*" OR "4-year institution" OR "community college*") |
| 3. TS=("natural science*" OR "natural resource*" OR "environmental science*" OR "environmental stud*" OR forest* OR wilderness OR biolog* OR bioscience* OR geolog* OR geoscience* OR ecolog* OR "earth science*" OR "atmospheric science*" OR "plant science*" OR botan* OR "animal science*" OR zoolog* OR ecolog* OR "marine science*" OR "agricultur*" OR farm OR farms OR farming OR "life science*" OR "soil science*" OR "crop science*" OR "pest management" OR entomolog* OR hydrolog* OR limnolog* OR mammalog* OR ornitholog* OR herpetolog* OR oceanograph* OR fisher* OR “plant pathology” OR sylvicultur* OR "ecosystem science*" OR conservation OR arboriculture OR landscape* OR watershed* OR geograph* OR bioscience* "food system*" OR paleontolog* OR paleobiolog* OR paleobotany) |
| 4. 1 AND 2 AND 3 AND Date filter (2000-present) |

#

#### **Table S2.** Definitions of common terms used within the scoping review.

| **Term** | **Definition** |
| --- | --- |
| Field | Occuring in the outdoors outside of a typical classroom* |
| Field Course | A credit-based, full-semester (or equivalent) class where students leave the classroom and learn outdoors and interact with the outdoor environment at least once (including summer session, winter session).* |
| Natural Sciences | Disciplines that deal with natural events (i.e., independent and dependent variables in nature) using scientific methods (Ledoux, 2002). |
| US-based Academic Institutions | Public or private educational institutions in the US (i.e., universities and colleges), offering for-credit programs at the bachelor’s or higher degree level.* |
| Undergraduate | A student at a college or university who is studying for their first degree, excluding continuing education students.* |
| Knowledge | Factual and conceptual understanding; metacognition; concept retention** |
| Affective Outcomes | Including values, motivations, identity, attitudes, self-efficacy, values, confidence, sense of place, emotion, experience, social connection, belonging.* |
| Behavioral Outcomes | Including participation and retention in the class, enrollment in other natural science classes, enrollment in other field courses, engagement in environmental extracurricular activities, applying for and earning a job in the natural sciences.* |
| Science-  Process Skills | Including, the scientific method, procedural learning, research design, data collection, data management, data analysis ** |
| Transferable Skills | Including, collaboration, communication, leadership, project management, presentation, organization, decision making, time management, problem solving* |
| Technical Skills | Field skills, field identification, mapping, standard survey methods*, outdoor skills |
| Critical Thinking | Reasoning, making judgments and decisions, and problem solving (Willingham, 2008), as well as higher order thinking, and metacognition (Ennis, 1998). |

**definition from scoping review protocol (verbatim)*

*** definition from data analysis notes (verbatim)*

#### **Table S3.** Data Extraction Categories, Study Design

| **Study Design** | **Data Extraction Categories** |
| --- | --- |
| Data Type | *Qualitative Quantitative Mixed Methods* |
| Student Outcome Data Types | *Course Assessments*  *Course Evaluations*  *Direct Observations*  *Field Notes/Reflections Focus Groups*  *Informal Discussions*  *Interviews*  *Surveys* |
| Study Design | *Control /Comparison Group Counterfactual Analysis Pre/Post Assessments* |
| Study Rigor (Methodological & Analytical) | *More Rigorous Moderately Rigorous Less Rigorous* |

#### **Table S4.** Data Extraction Categories, External Factors Affecting Participation

| **External Factors** | **Data Extraction Categories and Sources** |
| --- | --- |
| Institution Location | *[Designated by US Census Bureau*^1^*]* |
| Field Site Location | *Latitude & Longitude** |
| Public/Private University Status | *[National Center for Education Statistics designation*^2^*]* |
| Land Grant University Status | *[USDA National Institute for Agriculture designation*^3^*]* |
| Community College Status | *[American Association of Community Colleges designation*^4^*]* |
| Minority Serving Institution Status | *Historically Black Colleges and Universities*  *Hispanic Serving Institutes*  *Tribal Colleges and Universities*  *Asian American Serving Institutes*  *Native American Pacific Islander Serving Institutes* |

* If more than one or two field sites were described, we triangulated all the locations and chose the center point as the field site point to increase clarity on the map. We also used this centroid method for courses that mentioned multiple locations in one state but did not provide specific coordinates, in which case we took the center point of the state (i.e., Alabama and Montana). Four articles did not provide names and locations for the institution or the field site and were, therefore, removed from the analysis.

^1^ https://www2.census.gov/geo/pdfs/maps-data/maps/reference/us_regdiv.pdf

^2^ https://nces.ed.gov/collegenavigator/

^3^ https://www.nifa.usda.gov/land-grant-colleges-and-universities-partner-website-directory

^4^ https://www.aacc.nche.edu/college-finder/

#### **Table S5.** Data Extraction Categories, Internal Factors Affecting Participation*

| **Internal Factors** | **Data Extraction Categories** |
| --- | --- |
| Gender | *[as reported in text]* |
| Race/Ethnicity | *[as reported in text]* |
| First-Generation Status | *[as reported in text]* |
| Student Major | *[as reported in text]* |
| Class Standing | *Freshman / Sophomore / Junior / Senior* |
| Student Motivations | *[extracted and thematically coded from data]* |
| Student Barriers | *[extracted and thematically coded from data]* |
| Prior Field Experience | *[as reported in text]* |

*In the text, we use the term “minority and minoritized” to bring attention to the institutional processes that render individuals as minoritities rather than presuming their status based on prior or inherent identity.

#### **Table S6.** Data Extraction Categories, Field Course Design

| **Field Course Design** | **Data Extraction Categories** |
| --- | --- |
| Natural Science Discipline | *[NSF Divisions (NSF, n.d.)]* |
| Reported Study Area | [Habitat Classification Scheme, created by the International Union for Conservation of Nature (IUCN, n.d)] |
| Length of Time in Field | *[In days]* |
| Field Season | *Spring [Mar.-May]*  *Summer [June-Aug.]*  *Fall [Sept.-Nov.]*  *Winter [Dec.-Feb.]* |
| Reported Course Costs | *[in US Dollars]* |

#### **Table S7.** Data Extraction Categories, Student Outcomes

| **Student Outcomes** | **Data Extraction Categories** |
| --- | --- |
| Knowledge | *Concept Retention*  *Factual & Conceptual Understanding*  *Metacognition* |
| Affect | *Attitudes*  *Belonging*  *Confidence*  *Emotion*  *Experience*  *Identity*  *Self-efficacy*  *Sense of Place*  *Social Connection*  *Values* |
| Behavior | *Participation in Class*  *Retention in Class*  *Enrollment in other Natural Science Classes*  *Enrollment in other Field Courses* |
| Skill-Based Outcomes | *Science-process Skills (e.g. research skills)*  *Transferable Skills (e.g. collaboration and communication)*  *Technical Skills (e.g. mapping)*  *Critical Thinking* |

#### **Table S8.** Full citations of articles included in the systematic scoping review

| Abolins, M. (2014). Undergraduates discovering folds in “flat” strata: an unusual undergraduate geology field methods course. *Journal of Geoscience Education*, *62*(2), 264-277.  Ahn, C. (2015). K-12 Participation Is Instrumental in Enhancing Undergraduate Research and Scholarship Experience. *Journal of College Teaching & Learning*, *12*(2), 87-94.  Alagona, P. S., & Simon, G. L. (2010). The role of field study in humanistic and interdisciplinary environmental education. *Journal of Experiential Education*, *32*(3), 191-206.  Albrecht, G. L., Ketterings, Q. M., Czymmek, K. J., van Amburgh, M. E., & Fox, D. G. (2006). Whole farm nutrient management: Capstone course on environmental management of dairy farms. *Journal of Natural Resources and Life Sciences Education*, *35*(1), 12-23.  Anderson, K. (2009). Undergraduate horse industry study tour enhances experiential learning. *NACTA Journal*, 18-22.  Archie, T. (2007). *Examination of the value of community in natural resources education, An* (Doctoral dissertation, Colorado State University).  Atchison, C. L. (2011). *The significance of access: Students with mobility impairments constructing geoscience knowledge through field-based learning experiences* (Doctoral dissertation, The Ohio State University).  Balliet, R. N., Riggs, E. M., & Maltese, A. V. (2015). Students' problem solving approaches for developing geologic models in the field. *Journal of Research in Science Teaching*, *52*(8), 1109-1131.  Balliet, R. N. (2012). *Evaluation of undergraduate geologists' problem solving and cognition during field exams using a mixed methods approach* (Doctoral dissertation, Purdue University).  Bauerle, T. L., & Park, T. D. (2012). Experiential learning enhances student knowledge retention in the plant sciences. *HortTechnology*, *22*(5), 715-718.  Baum, S. D., Aman, D. D., & Israel, A. L. (2012). Public Scholarship Student Projects for Introductory Environmental Courses. *Journal of Geography in Higher Education*, *36*(3), 403-419.  Brackney, D. L. (2008). *Influence of field study on learning and attitudes toward science*. (Doctoral Dissertation, Wayne State University).  Brannstrom, C., & Houser, C. (2015). “Riding the rip”: an experiential and integrated human–physical geography curriculum in Costa Rica. *Journal of Geography in Higher Education*, *39*(4), 527-542.  Brenner, J. C., Hamilton, J. G., Stork, A., Jordan, J., & Drake, T. (2017). Addressing Estrangement from Nature with a Night Class in the Forest. *Case Studies in the Environment*.  Carpenter, D. M. (2008). *Influencing attitudes toward science through field experiences in biology*. (Doctoral Dissertation, The University of Alabama).  Collins, C. R., & Donahue, L. (2019). Improving Eco-Literacy through Service Learning: A Natural History Service Project Case Study. *The American Biology Teacher*, *81*(4), 222-227.  Colón, C. P. (2016). Addressing civic issues in biology lab through citizen science. In *Civic Engagement Pedagogy in the Community College: Theory and Practice* (pp. 171-187). Springer, Cham.  Driscoll, B. A. (2011). *Graduates' perspectives regarding the impact of the integration of experiential learning in academic programs*. (Master’s Thesis, Iowa State University).  Dykas, M. J., & Valentino, D. W. (2016). Predicting performance in an advanced undergraduate geological field camp experience. *Journal of Geoscience Education*, *64*(4), 314-322.  Elkins, J., Elkins, N. M., & Hemmings, S. N. (2008). GeoJourney: A field-based, interdisciplinary approach to teaching geology, Native American cultures, and environmental studies. *Journal of College Science Teaching*, *37*(3), 18.  Elwood, S. (2009). Integrating participatory action research and GIS education: Negotiating methodologies, politics and technologies. *Journal of Geography in Higher Education*, *33*(1), 51-65.  Enos-Berlage, J. (2012). Development of a water-quality lab that enhances learning & connects students to the land. *The American Biology Teacher*, *74*(7), 471-478.  Feig, A. D. (2010). Technology, accuracy and scientific thought in field camp: An ethnographic study. *Journal of Geoscience Education*, *58*(4), 241-251.  Flaherty, E. A., Walker, S. M., Forrester, J. H., & Ben‐David, M. (2017). Effects of course‐based undergraduate research experiences (CURE) on wildlife students. *Wildlife Society Bulletin*, *41*(4), 701-711.  Gibson, K. D., Benjamin, T. J., Oseto, C. Y., & Adams, M. M. (2012). A short-term study abroad course in Costa Rica. *NACTA Journal*, *56*(1), 23.  Gillie, L., & Bizub, A. L. (2012). In Darwin's Footsteps: An On and Off-Campus Approach to Teaching Evolutionary Theory and Animal Behavior. *Bioscene: Journal of College Biology Teaching*, *38*(1), 15-21.  Godfrey, C. M., Barrett, B. S., & Godfrey, E. S. (2011). Severe weather field experience: An undergraduate field course on career enhancement and severe convective storms. *Journal of Geoscience Education*, *59*(3), 111-118.  Gonzales, D., & Semken, S. (2009). A comparative study of ﬁeld inquiry in an undergraduate petrology course. *Field geology education: Historical perspectives and modern approaches: Geological Society of America Special Paper*, *461*, 205-221.  Goralnik, L., Thorp, L., & Rickborn, A. (2018). Food system field experience: STEM identity and change agency for undergraduate sustainability learners. *Journal of Experiential Education*, *41*(3), 312-328.  Higley, C. A. (2019). *Affective Education by Design: An Experiential Pedagogy for Natural Resources Education*. (Doctoral Dissertation, Michigan State University).  Hill, H. M., & Karlin, M. (2019). Reflections on an international research immersion field study as a high impact practice to produce publishable papers by underrepresented undergraduates. *Frontiers in psychology*, *10*, 601.  Hoalst-Pullen, N., & Gatrell, J. D. (2011). Collaborative learning and interinstitutional partnerships: An opportunity for integrative fieldwork in geography. *Journal of Geography*, *110*(6), 252-263.  Houser, C., Brannstrom, C., Quiring, S. M., & Lemmons, K. K. (2011). Study abroad field trip improves test performance through engagement and new social networks. *Journal of Geography in Higher Education*, *35*(4), 513-528.  Hudak, P. E. (2003). Campus field exercises for introductory geoscience courses. *Journal of Geography*, *102*(5), 220-22.  Kamen, E., & Leri, A. (2019). Promoting STEM Persistence Through an Innovative Field Trip–Based First-Year Experience Course. *Journal of College Science Teaching*, *49*(2), 24-33.  Kelley, D. F., Uzunlar, N., Lisenbee, A., Beate, B., & Turner, H. E. (2017). A Capstone Course in Ecuador: The Andes/Galápagos Volcanology Field Camp Program. *Journal of Geoscience Education*, *65*(3), 250-262.  Kirkby, K. C. (2014). Place in the city: Place-based learning in a large urban undergraduate geoscience program. *Journal of Geoscience Education*, *62*(2), 177-186.  Koretsky, C. M., Petcovic, H. L., & Rowbotham, K. L. (2012). Teaching environmental geochemistry: An authentic inquiry approach. *Journal of Geoscience Education*, *60*(4), 311-324.  Lo, C. P., Affolter, J. M., & Reeves, T. C. (2002). Building environmental literacy through participation in GIS and multimedia assisted field research. *Journal of Geography*, *101*(1), 10-19.  Lysne, S. J., & Miller, B. G. (2015). Using mobile devices to engage students in evolutionary thinking. *The American Biology Teacher*, *77*(8), 624-627.  MacLaren, R. D., Schulte, D., & Kennedy, J. (2012). Field Research Studying Whales in an Undergraduate Animal Behavior Laboratory. *Bioscene: Journal of College Biology Teaching*, *38*(1), 3-10.  Mapp, K. J. (2015). *Science and Ecological Literacy in Undergraduate Field Studies Education* (Doctoral dissertation, University of Wyoming. Libraries).  Marshall, J. S., Gardner, T. W., Protti, M., & Nourse, J. A. (2009). International geosciences ﬁeld research with undergraduate students: Three models for experiential learning projects investigating active tectonics of the Nicoya Peninsula, Costa Rica. *Field Geology Education: Historical Perspectives and Modern Approaches*, *461*, 77.  Mason, N. A., Brunner, R. M., Ballen, C. J., & Lovette, I. J. (2018). Cognitive and Social Benefits among Underrepresented First-Year Biology Students in a Field Course: A Case Study of Experiential Learning in the Galápagos. *Frontiers: The Interdisciplinary Journal of Study Abroad*, *30*(3), 1-19.  McKim, B. R., Latham, L., Treptow, E., & Rayfield, J. (2013). A repeated measures study of the short-term influences of high-impact practices on college students' learning styles. *NACTA Journal*, *57*(3a), 122.  McLaughlin, J., Patel, M., Johnson, D. K., & de la Rosa, C. L. (2018). The Impact of a Short-Term Study Abroad Program That Offers a Course-Based Undergraduate Research Experience and Conservation Activities. *Frontiers: The Interdisciplinary Journal of Study Abroad*, *30*(3), 100-118.  McLaughlin, J. S. (2010). Reimagining science education and pedagogical tools: blending research with teaching. *Educause Quarterly*, *33*(1), n1.  Mroz, E. A. (2015). *Environmental attitudes and connections to landscapes of undergraduate regional field course students* (Doctoral dissertation, Montreat College).  Mullens, J. B. (2016). Student perceptions of a recreation trail assignment as a valuable learning experience for Geography undergraduates. *Journal of Geography*, *115*(6), 244-255.  Odom, S. F., Shehane, M., Moore, L. L., & McKim, B. (2014). An Analysis of a High-Impact Field Experience in Agriculture: Documenting Critical Thinking Skills through Reflection. *NACTA Journal*, *58*(3).  Paradis, T. W., & Dexter, L. R. (2007). Learner-centered teaching and assessment in an undergraduate field analysis course. *Journal of Geography*, *106*(4), 171-180.  Rathburn, S. L., & Weinberg, A. E. (2011). Undergraduate student satisfaction and achievement at the GetWET Observatory: A fluid learning experience at Colorado State University. *Journal of Geoscience Education*, *59*(2), 47-55.  Schiappa, T. A., & Smith, L. (2019). Field experiences in geosciences: A case study from a multidisciplinary geology and geography course. *Journal of Geoscience Education*, *67*(2), 100-113.  Simmons, M. E., Wu, X. B., Knight, S. L., & Lopez, R. R. (2008). Assessing the influence of field-and GIS-based inquiry on student attitude and conceptual knowledge in an undergraduate ecology lab. *CBE—Life Sciences Education*, *7*(3), 338-345.  Steiger, R., Abegg, B., & Jänicke, L. (2016). Rain, rain, go away, come again another day. Weather preferences of summer tourists in mountain environments. *Atmosphere*, *7*(5), 63.  Stumpf, R. J., Douglass, J., & Dorn, R. I. (2008). Learning desert geomorphology virtually versus in the field. *Journal of Geography in Higher Education*, *32*(3), 387-399.  Todd, C. E. D., & Goeke, E. R. (2012). Incorporating student-led field trips and learner-centered teaching in a capstone geology course. *Journal of Geoscience Education*, *60*(3), 268-276.  Vogt, B. J., & Skop, E. (2017). The Silverton field experience: a model geography course for achieving high-impact educational practices (HEPs). *Journal of Geography in Higher Education*, *41*(4), 574-589.  Warkentin, T. (2011). Cultivating urban naturalists: Teaching experiential, place-based learning through nature journaling in Central Park. *Journal of Geography*, *110*(6), 227-238.  Wilson, M. (2012). *Utilizing an Artificial Outcrop to Scaffold Learning Between Laboratory and Field Experiences in a College-Level Introductory Geology Course*. (Doctoral Dissertation, Arizona State University).  Wilson, H., Leydon, J., & Wincentak, J. (2017). Fieldwork in geography education: Defining or declining? *The state of fieldwork in Canadian undergraduate geography programs. Journal of Geography in Higher Education*, 41(1), 94-105.  Winn, W., Stahr, F., Sarason, C., Fruland, R., Oppenheimer, P., & Lee, Y. L. (2006). Learning oceanography from a computer simulation compared with direct experience at sea. *Journal of Research in Science Teaching*, *43*(1), 25-4 |
| --- |

#### **Table S9.** Compilation of all the majors listed as participating in US-based field courses

| Agricultural Business  Agricultural Science  Agricultural Systems Technology  Agronomy  Animal Ecology  Animal Science  Anthropology  Atmospheric Science  Biochemistry  Biology  Biology Management  Business Administration  Communications  Criminal justice  Crop science  Economics  Education  Entomology  Environmental geosciences  Environmental sciences  Food Science  Genetics  Geochemistry  Geography  Geology  GISc  Horticulture  Insect Science  Landscape Architecture  Microbiology  Nursing  Plant Science  Political Science  Psychology  Science and Non-science  Sociology  Soil Science  Wildlife and Fisheries  Zoology |
| --- |

#### **Table S10.** Validated assessments cited in included articles, sorted by student outcomes.

| **Thematic outcomes** | **Specific outcome** | **Validated assessment name** | **Assessment citation** |
| --- | --- | --- | --- |
| Knowledge | Knowledge | Climate change perceptions | (Leiserowitz, et al. 2010) |
|  |  | Geoscience Concept Inventory | (Libarkin & Anderson, 2006) |
|  |  | Kolb Learning Style Inventory | (Kolb, 2007) |
| Affect | Attitudes toward course | Science Laboratory Environmental Inventory | (West, 2003) |
|  | Attitudes toward science | Interest in Research | (Bishop & Bieschke, 1998) |
|  | Attitudes toward science | Attitudes toward science | (Harsh, Maltese, & Tai. 2012) |
|  | Confidence | College Self Efficacy Inventory | (Solberg et al., 1993) |
|  |  | New General Self Efficacy Scale | (Chen et al., 2001) |
|  | Connection to environment | Place Attachment Instrument | (Semken, & Freeman, 2008) |
|  |  | New Ecological Paradigm model | (Dunlap, Van Liere, Mertig, & Jones, 2000) |
|  | Motivation | Science Motivation Questionnaire (SMQ) | (Glynn & Koballa, 2006) |
| Skill-based outcomes | Critical thinking | Test on Scientific Literacy Skills | (Gormally, et al., 2012) |
